# Role of Sortase A in *Lactobacillus gasseri* Kx110A1 Adhesion to Gastric Epithelial Cells and Competitive Exclusion of *Helicobacter pylori*

**DOI:** 10.3389/fmicb.2019.02770

**Published:** 2019-12-03

**Authors:** Fanglei Zuo, Amulya Appaswamy, Hanna G. Gebremariam, Ann-Beth Jonsson

**Affiliations:** Department of Molecular Biosciences, The Wenner-Gren Institute, Stockholm University, Stockholm, Sweden

**Keywords:** sortase, sortase-dependent protein, *lactobacillus*, *helicobacter pylori*, host cell adhesion, competitive exclusion

## Abstract

We have previously shown that *Lactobacillus gasseri* Kx110A1, a human stomach isolate, can colonize mouse stomach and reduce the initial colonization of *Helicobacter pylori*. Here, we investigated the role of sortase-dependent proteins (SDPs) involved in these functions by the construction of a mutant for *srtA*, the gene encoding the housekeeping sortase that covalently anchors SDPs to the cell surface. The *srtA* mutant showed a decrease in hydrophobicity and autoaggregation under acidic conditions, indicating the effect of SDPs on cell surface properties. Correspondingly, the *srtA* mutant lost the capacity to adhere to gastric epithelial cells, thus resulting in an inability to provide a physical barrier to prevent *H. pylori* adherence. These results indicate that sortase A is a key determinant of the cell surface properties of *L. gasseri* Kx110A1 and contributes to *Lactobacillus*-mediated exclusion of *H. pylori*. Understanding the molecular mechanisms by which lactobacilli antagonize *H. pylori* might contribute to the development of novel therapeutic strategies that take advantage of health-promoting bacteria and reduce the burden of antibiotic resistance.

## Introduction

Stomach cancer is the fifth most frequently diagnosed cancer and the third leading cause of cancer death, with over 10,00,000 new cases in 2018 and an estimated 783,000 deaths. *Helicobacter pylori* is the main risk factor for developing stomach cancer, with almost 90% of new cases of noncardia gastric cancer attributed to this bacterium (Bray et al., 2018). Standard triple therapy, using a proton pump inhibitor, combined with two antibiotics (amoxicillin plus clarithromycin, metronidazole, or a fluoroquinolone), is highly recommended as the first-line treatment but now provides unacceptably low treatment success owing to the increase in antibiotic-resistant *H. pylori* infections (Qureshi et al., 2019). Recently, the WHO published a list of bacteria for which new antibiotics are urgently needed, and clarithromycin-resistant *H. pylori* was included in the high priority group (Tacconelli and Magrini, 2017). However, antibiotic-based therapy changes the composition of the intestinal microbiota, causes side effects, and in particular, encourages widespread antibiotic resistance (Nord et al., 1984; Qureshi et al., 2019). Hence, an alternative or adjunct therapy is urgently needed to increase the eradication rate of *H. pylori* and reduce the use of antibiotics.

The human gastrointestinal tract, including the harsh environment of the stomach, harbors a great variety of bacteria, of which *Lactobacillus* species are prominent members. The microbiota plays a key role in controlling the colonization of pathogens and preventing infections. Some *Lactobacillus* strains have been developed as probiotics that are widely used as adjunct treatments to antibiotics for the eradication of *H. pylori* (Zhang et al., 2015; Goderska et al., 2018). Lactobacilli can combat *H. pylori* through multiple strategies, including competitive adhesion, production of antimicrobial substances, coaggregation, immunomodulation, improvement of the epithelial barrier integrity, and inhibition of virulence gene expression (Qureshi et al., 2019). Lactobacilli are effective in eradicating the pathogen and are also responsible for reduced side effects due to antibiotic therapy (Zhang et al., 2015). However, the molecular mechanisms underlying the antagonistic effect of lactobacilli against *H. pylori* are still largely unknown, partly due to insufficient genetic tools, especially mutagenesis methods.

Lactobacilli possess shared probiotic mechanisms among species in which the cell surface-associated sortase-dependent proteins (SDPs) play an important role in bacteria-host interactions (Call and Klaenhammer, 2013; Sanders et al., 2018). SDPs contain a conserved C-terminal cell wall sorting motif, generally LPXTG, and are linked to the peptidoglycan layer by the housekeeping sortase, sortase A (SrtA). Sortase A and SDPs have been studied in various *Lactobacillus* species in adhesion to epithetical cells (van Pijkeren et al., 2006; Munoz-Provencio et al., 2012; Malik et al., 2013; Jensen et al., 2014), mucus barrier maintenance (van Pijkeren et al., 2006), biofilm formation (Velez et al., 2010; Malik et al., 2013), and immunomodulation (von Schillde et al., 2012; Remus et al., 2013; Call et al., 2015). However, the role of sortase A and SDPs in *Lactobacillus*-mediated pathogen antagonization is rarely reported (Souza et al., 2017).

In a previous study, we showed that a human stomach isolate, *Lactobacillus gasseri* Kx110A1, is able to colonize the mouse stomach and reduce the initial colonization of *H. pylori* (de Klerk et al., 2016). Here, we investigated the role of SDPs in *L. gasseri* Kx110A1 adhesion to gastric epithelial cells and inhibition of *H. pylori* adherence by the construction of sortase A-deficient derivatives.

## Materials and Methods

### Bacterial Strains, Media, and Growth Conditions

The bacterial strains and plasmids used in this study are listed in Table 1*. Lactobacillus* strains were grown on Rogosa agar plates and cultured overnight in deMan Rogosa Sharpe (MRS) broth (Oxoid) at 37°C and 5% CO_2_ in a humidified environment. *Helicobacter pylori* strain 67:21 (Björkholm et al., 2001) was grown on Columbia blood agar plates (Acumedia) supplemented with 8% defibrinated horse blood and 8% inactivated horse serum (Håtunalab) for 3 days at 37°C under microaerophilic conditions, i.e., in an incubator with 5% O_2_, 10% CO_2_, and 85% N_2_. *Escherichia coli* strains were cultured in Luria-Bertani broth at 37°C with rotary shaking at 200 rpm or on LB agar plates. When needed, antibiotics were supplemented at the following concentrations: 10 μg/ml chloramphenicol for *Lactobacillus* strains and the *E. coli* VE7108 strain, 100 μg/ml ampicillin for the *E. coli* DH5α strain, and 50 μg/ml bacitracin for *H. pylori*.

**Table 1:** Bacterial strains and plasmids used in this study.

Strains or plasmids	Characteristics^a^	Source
**Strains**		
*E. coli* DH5α	*fhuA2 Δ(argF-lacZ)U169 phoA glnV44 Φ80 Δ(lacZ)M15 gyrA96 recA1 relA1*	Invitrogen
*E. coli* VE7108	Km^r^, host of pNZ8048 and pINTZrec	Mora et al. (2004)
*L. gasseri* Kx110A1	Isolated from human gastric	de Klerk et al. (2016)
*L. gasseri* Kx110A1 *ΔsrtA*	*L. gasseri* Kx110A1 *srtA* deletion mutant	This study
*L. gasseri* Kx110A1 *ΔsrtA/srtA*	*L. gasseri* Kx110A1 *srtA* deletion mutant harboring pNZ8048-comSrtA	This study
**Plasmids**		
pUC18	Amp^r^, *E. coli* cloning vector	Thermo Scientific
pUC18-∆srtA	Amp^r^, pUC18 derivative containing upstream and downstream DNA of *L. gasseri* Kx110A1 *srtA* gene	This study
pINTZrec	Cm^r^, IPSD plasmid used to assist bacterial recombineering	Zuo et al. (2019)
pINTZrec-∆srtA	Cm^r^, pINTZrec derivative containing upstream and downstream DNA of *L. gasseri* Kx110A1 *srtA* gene	This study
pNZ8048	Cm^r^, SH71 replicon plasmid	De Ruyter et al. (1996)
pNZ8048-comSrtA	Cm^r^, pNZ8048 derivative containing native *srtA* expression cassette	This study

a *Km^r^, kanamycin resistant; Cm^r^, chloramphenicol resistant; Amp^r^, ampicillin resistant*.

### Epithelial Cell Lines and Culture Conditions

The gastric epithelial cell line AGS (ATCC CRL-1739) was cultured in RPMI 1640 (Life Technologies) supplemented with 10% heat-inactivated fetal bovine serum (Sigma-Aldrich). The cells were maintained at 37°C and 5% CO_2_ in a humidified environment. The cells were seeded in tissue culture plates, the day before the experiment to form a monolayer overnight.

### DNA Manipulations

Plasmid DNA of *E. coli* was extracted using an E.Z.N.A. Plasmid DNA Mini kit I (Omega, USA). *L. gasseri* DNA was isolated using a Wizard^®^ Genomic DNA Purification kit (Promega, USA). Primers were synthesized by Eurofins Genomics (Ebersberg, Germany). Phusion High-Fidelity DNA polymerase (Thermo Fisher Scientific, Finland) was used for the amplification of *L. gasseri* genomic DNA, and GoTaq DNA polymerase (Promega, USA) was used for colony PCR. The PCR products were purified by a Zymoclean™ Gel DNA Recovery kit (Zymo Research, USA). The restriction endonucleases and T4 DNA ligase were obtained from Thermo Scientific. All of the procedures were conducted according to the manufacturer’s instructions.

### Identification of Sortase A Gene and the Substrates of *srtA*-Encoded Protein

The sortase A-encoding gene *srtA* (corresponding to *LGAS_0825* of *L. gasseri* ATCC33323) was amplified from *L. gasseri* Kx110A1 genomic DNA by primers srtA-F/srtA-R and sequenced. To check for the presence of possible substrates of sortase A, primers were designed according to the gene sequences of reported SDPs in *L. gasseri* ATCC33323 and SBT2055 (Supplementary Table S1). Appropriately sized PCR products were considered positive. The PCR program was as follows: initial denaturation at 95°C for 5 min, followed by amplification for 30 cycles with denaturation at 95°C for 30 s; annealing at 54°C for 20 s; and extension at 72°C for 30 s.

### Construction of an *L. gasseri srtA* Mutant

The primers used in this study are listed in Supplementary Table S1. A *srtA* gene deletion mutant of *L. gasseri* Kx110A1 was generated by IPSD-assisted genome engineering (Zuo et al., 2019). Two 975 bp DNA fragments, upstream and downstream of the *srtA* gene, were amplified from the genomic DNA of *L. gasseri* Kx110A1 using the primer pairs srtA-up-F/srtA-up-R and srtA-down-F/srtA-down-R, respectively. The PCR program was as follows: initial denaturation at 95°C for 5 min, followed by amplification for 30 cycles with denaturation at 95°C for 30 s; annealing at 56°C for 20 s; and extension at 72°C for 60 s. The upstream DNA fragment was inserted into the pUC18 vector between *Sac*I and *BamH*I to generate pUC18-srtA-up. Then, the downstream DNA fragment was inserted into pUC18-srtA-up between *BamH*I and *Sph*I to generate pUC18-∆srtA. pUC18-∆srtA was digested with *Sac*I and *Sph*I, and the ∆srtA fragment was inserted into a similarly digested pINTZrec vector to generate pINTZrec-∆srtA. The pINTZrec-∆srtA plasmid was transformed into *L. gasseri* Kx110A1 by electroporation as described previously (De Keersmaecker et al., 2006). The transformants were confirmed by colony PCR followed by PCR on DNA extracted from pure cultures.

Single colonies of *L. gasseri* Kx110A1 harboring the plasmid pINTZrec-∆srtA were grown overnight in MRS broth containing 10 μg/ml chloramphenicol. The cultures were inoculated (1%, v/v) into MRS broth without antibiotics and grown at 37°C until the OD_600 nm_ reached ~ 0.30, then supplemented with 100 ng/ml sakacin P (SppIP) (Genscript). The cultures were allowed to grow overnight, and serial dilutions were plated on MRS agar supplemented with 10 μg/ml chloramphenicol and 100 ng/ml SppIP. The single-crossover events were detected by colony PCR followed by PCR using primer pairs srtAseq-F/pIrecSC-R or pIrecSC-F/srtAseq-R on DNA extracted from pure cultures. For double-crossover selection, the single-crossover clones were grown overnight in MRS broth in the absence of antibiotics, serial dilutions of which were subsequently spread on MRS agar. The colonies from the MRS agar were replicated on MRS agar containing 10 μg/ml chloramphenicol, and the Cm^s^ colonies were selected and detected by PCR using the primer pair srtAseq-F/srtAseq-R on extracted DNA. The double-crossover *srtA* deletion mutant was selected according to the correct size of the PCR product. The final *srtA* mutant was confirmed by PCR and sequencing.

### Complementation

To complement the *srtA* deletion in *L. gasseri* Kx110A1, the *srtA* native expression cassette was amplified using the primer pair comSrtA-F/comSrtA-R, digested with *Bgl*II and *Xho*I, and ligated into similarly digested pNZ8048 to generate pNZ8048-comSrtA. The pNZ8048-comSrtA plasmid was electroporated into *L. gasseri* Kx110A1-ΔsrtA to generate the sortase A complemented strain *L. gasseri* Kx110A1-ΔsrtA/srtA. The transformants were confirmed by colony PCR followed by PCR on DNA extracted from pure cultures. The final complemented strain was confirmed by PCR and sequencing.

### Microbial Adhesion to Solvents

The assay for Microbial Adhesion to Solvents (MATS) was performed according to Kos et al. (2003) with some modifications. Three different solvents, including xylene, chloroform, and ethyl acetate, which represent either nonpolar (hydrophobic), monopolar, and acidic (electron donor), or monopolar and basic (electron acceptor) surfaces, respectively, were tested in this study. Overnight cultures were harvested by centrifugation at 4,500 rpm for 5 min and washed twice with PBS pH 7.4. The pellet was resuspended in the same buffer at OD_600_ = 1.0. Two milliliters of bacterial suspension and 0.4 ml of solvent was mixed by vortexing for 120 s. The aqueous phase was carefully transferred after 20 min of incubation at room temperature, and the absorbance was measured at 600 nm. The percentage of microbial adhesion to the solvent was calculated as (1 − *A_1_*)/*A_0_* × 100, where *A_0_* and *A_1_* are the absorbances before and after extraction with solvents, respectively.

### Autoaggregation and Coaggregation Assays

*L. gasseri* autoaggregation and coaggregation with *H. pylori* was assessed as previously described (Kos et al., 2003) with some modifications. Briefly, *L. gasseri* strains were grown for 16 h at 37°C in MRS broth, the cells were harvested by centrifugation at 4,500 rpm for 5 min, and the pellets were washed twice and suspended in PBS (pH 7.4 or pH 4.2) at OD_600_ = 1.0. Autoaggregation was determined after 3 h of incubation without agitation at 37°C. A total of 0.1 ml of the upper suspension was transferred to another tube with 0.9 ml of PBS, and the absorbance (*A*) was measured at 600 nm. The autoaggregation percentage was expressed as 1 − (*A_t_*/*A_0_*) × 100, where *A_t_* represents the absorbance at time *t* = 3 h and *A_0_* represents the absorbance at *t* = 0 h. The preparation of cell suspensions for coaggregation was the same as that for the autoaggregation assay. Equal volumes (1 ml) of the cell suspension of *L. gasseri* and *H. pylori* were mixed and incubated without agitation at 37°C. The absorbance was determined for the mixture and for the bacterial suspensions alone. The coaggregation percentage was calculated as [(*A_x_* + *A_y_*) − 2 × *A_mix_*]/(*A_x_* + *A_y_*) × 100, where *A_x_* and *A_y_* represent the absorbance of the mixed bacterial suspensions at time 0 h, and *A_mix_* represents the absorbance of the mixed bacterial suspension after 3 h of incubation. The coaggregates were also observed by Gram staining and live-cell microscopy (Axiovert Z1, Zeiss).

### Adherence Assay

*H. pylori* from plates and *Lactobacillus* strains from overnight cultures were suspended to homogeneity in RPMI 1640. Epithelial cells in 48-well plates were infected with lactobacilli alone or together with *H. pylori* at an MOI of 100 for each species for 2 h. In the competition assay, lactobacilli and *H. pylori* were added simultaneously. In the exclusion assay, lactobacilli were preincubated with AGS cells for 2 h followed by washing to remove unbound bacteria before the addition of *H. pylori*. In the displacement assay, *H. pylori* were preincubated with AGS cells for 2 h followed by washing to remove unbound bacteria before the addition of lactobacilli. After incubation, the cells were washed three times with PBS to remove unbound bacteria. The host cells were lysed by treatment with 1% (m/v) saponin in RPMI 1640 for 5 min. The number of adhered CFUs was determined by serial dilution and spreading of the lysate on agar plates. *H. pylori* on blood agar plates supplemented with 50 μg/ml bacitracin were incubated for 5–7 days, and Rogosa plates with lactobacilli were incubated for 2 days.

### Quantitative PCR Analysis

The wild type *L. gasseri* Kx110A1 and its derivatives that had been incubated in RPMI 1640 medium for 2 h were resuspended in lysis buffer (30 mM Tris-HCl, 1 mM EDTA, 30 mg/ml lysozyme, 500 U/ml mutanolysin, and proteinase K; pH 8.0) and incubated for 20 min at room temperature, with 10 s of vortexing and 2 min rest cycles. The RNA was isolated using an RNeasy kit (Qiagen) according to the manufacturer’s instructions. SuperScript VILO Mastermix (Invitrogen) was used to synthesize the cDNA. Quantitative PCR (qPCR) was performed using a LightCycler 480 (Roche) and a SYBR Green I Master kit (Roche). The primers used are listed in Supplementary Table S1. The PCR program was as follows: initial denaturation at 95°C for 10 min, followed by amplification for 40 cycles with denaturation at 95°C for 10 s; annealing at 54°C for 20 s; and extension at 72°C for 20 s. The expression was normalized to that of the housekeeping gene *gyrB* encoding DNA gyrase B (Zhao et al., 2011). The expression levels were given as the fold change relative to the control samples.

### Nucleotide Sequence Accession Number

The complete nucleotide sequence of *srtA* of *L. gasseri* Kx110A1 and its flanking regions was submitted to NCBI and is available under accession number MK948931.

### Statistical Analysis

GraphPad Prism 7 was used for all statistical analyses. Significant differences were determined by one-way analysis of variance (ANOVA) followed by Dunnett’s multiple-comparisons test. Differences with *p* less than 0.05 were considered significant.

## Results

### Sortase A and Sortase-Dependent Proteins in *L. gasseri* Kx110A1

Since the fully sequenced genome of *L. gasseri* Kx110A1 is unavailable, we designed primers based on the genes encoding sortase A and SDPs in two well-studied *L. gasseri* strains, ATCC33323 and SBT2055 (Supplementary Table S1) (Kleerebezem et al., 2010; Arai et al., 2016). Amplification and sequencing of the *srtA* gene of *L. gasseri* 110A1 revealed a gene encoding 229 amino acids that shares 97% identity with *srtA* of *L. gasseri* ATCC33323. Furthermore, we identified 13 putative SDPs in the genome of *L. gasseri* Kx110A1 by using PCR, all of which are possible substrates of sortase A. Among these putative SDPs are five putative adhesion exoproteins (Supplementary Table S2).

### Construction of a Sortase A Knockout Mutant and Complemented Strain

**Figure 1:**
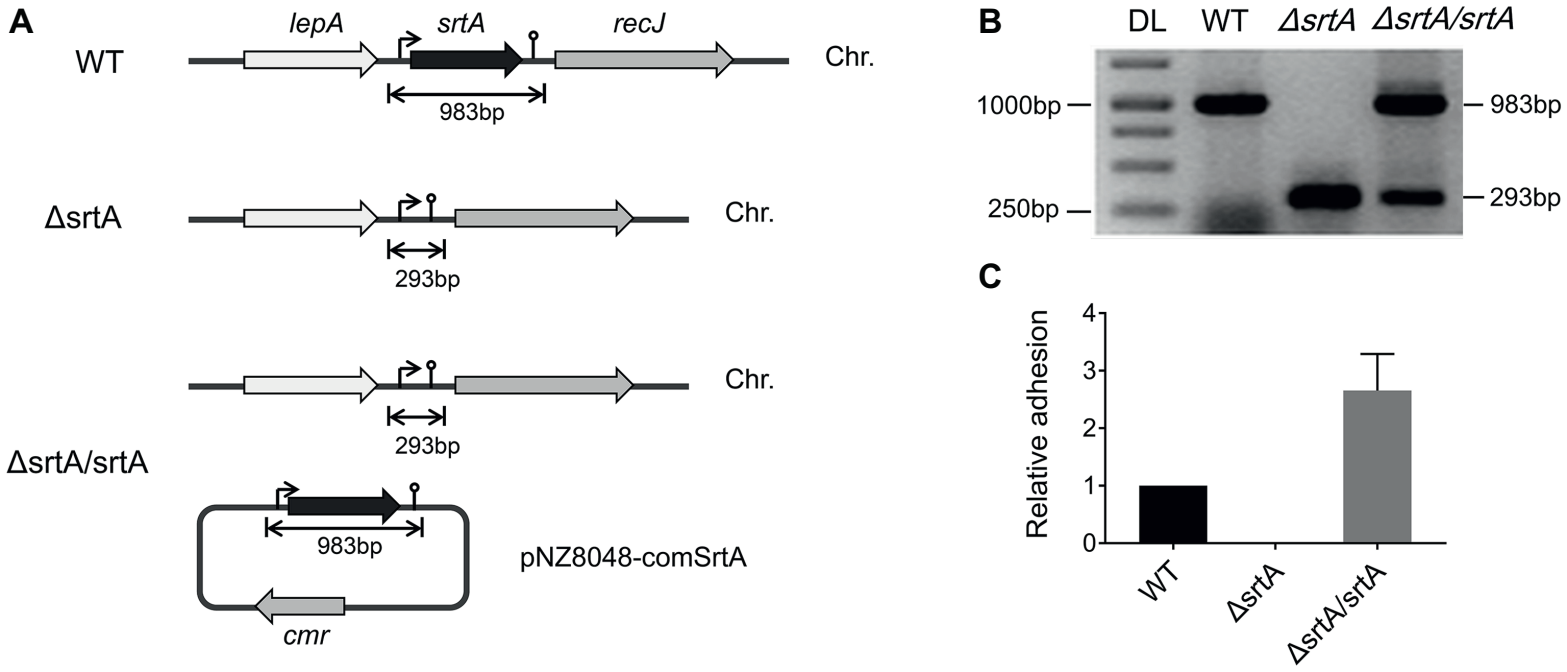
Generation of a *srtA* deletion mutant (∆srtA) and a complemented strain (∆srtA/srtA) in *L. gasseri* Kx110A1 (WT). **(A)** Schematic diagram of *srtA* inactivation in *L. gasseri* and generation of a complemented strain. The *lepA*, gene encoding the GTP-binding protein LepA; *srtA*, gene encoding sortase A; *recJ*, gene encoding exonuclease RecJ; *cmr*, chloramphenicol resistance gene; and Chr, chromosomal DNA. **(B)** PCR products separated by agarose gel electrophoresis. The *srtA* mutant and complemented strain were analyzed and verified by PCR using the primer pair comSrtA-F/R. DL, DNA ladder. **(C)** The *srtA* transcript level quantified by qPCR using the primer pair srtArt-F/R.

We generated a *srtA* deletion mutant (*ΔsrtA*) in *L. gasseri* Kx110A1 by double-crossover homologous recombination. A complemented strain (*ΔsrtA/srtA*) was constructed by introducing a native *srtA* expression cassette in plasmid pNZ8048 following transformation into the *srtA* mutant (Figure 1A). The constructs were confirmed by PCR and sequencing (Figure 1B). The transcript level of *srtA* in the wild type and its genetically modified derivatives was checked by qPCR, and the result showed that the mutant lacked *srtA* transcripts, while the transcript level of *srtA* in the complemented strain was restored (Figure 1C). Due to the deficiency of SrtA, the *srtA* mutant was also expected to lack the surface display of the predicted SDPs (Remus et al., 2013; Call et al., 2015). The transcript levels of the neighboring genes, *lepA* and *recJ* (Figure 1A), were not affected in the sortase A-deficient mutant (Supplementary Figure S1). These results suggest that the deletion of *srtA* did not cause any polar effects on the adjacent genes. Notably, the transcription of genes encoding the 13 putative SDPs (see Supplementary Table S2) was not affected by the *srtA* deletion (Supplementary Figure S2).

### Sortase A Affects the Cell Surface Properties of *L. gasseri* Kx110A1

**Figure 2:**
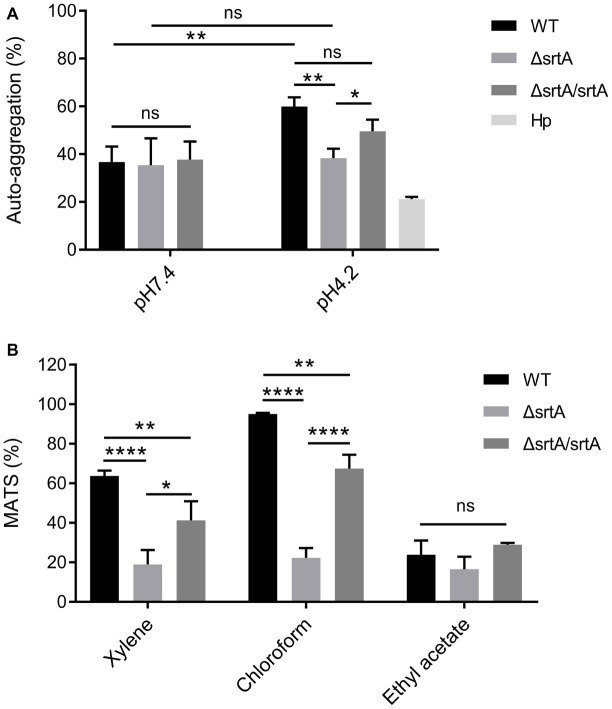
Cell surface properties of *L. gasseri* Kx110A1 and its mutant derivatives. **(A)** Autoaggregation ability of the WT and its derivatives. The overnight cultures were washed and resuspended in PBS pH 7.4 or PBS pH 4.2 at an OD_600_ of 1.0 and then incubated at 37°C for 3 h. **(B)** Hydrophobicity, electron donor, and electron acceptor characteristics of *L. gasseri* Kx110A1 and its derivatives. Data represent the means ± SD of three independent experiments, ^*^p < 0.05, ^**^p < 0.01, ^****^p < 0.0001, ns, no significance. Hp, *H. pylori*.

The growth rate of *ΔsrtA* in MRS broth was slightly but significantly slower than that of the wild type and complemented strain *ΔsrtA/srtA* (Supplementary Figure S3A), suggesting that deletion of *srtA* may lead to growth defects in *L. gasseri* Kx110A1. The overnight culture of the mutant strain *ΔsrtA* showed less cell sediment and more turbid upper suspension compared to that of the wild type (Supplementary Figure S3B). In addition, the single colonies of *ΔsrtA* on Rogosa plates were generally more spread out and thus larger than wild type colonies (Supplementary Figure S3C), suggesting a change in the autoaggregation of the sortase A-deficient mutant. The autoaggregation phenotype of the complemented strain *ΔsrtA/srtA* was restored to that of the wild type strain (Supplementary Figures S3B,C). The autoaggregation was further quantified by sedimentation assays for 3 h using PBS at pH 7.4. However, no difference was observed between the wild type and its derivatives (Figure 2A). During sedimentation assays overnight (Supplementary Figure S3B), the culture suspension reached pH values between 4.1 and 4.4. To investigate whether the aggregation ability is pH-dependent, we repeated the experiment using PBS at pH 4.2. The autoaggregation ability of the *srtA* mutant was significantly reduced compared to that of the wild type strain at pH 4.2 (*p* < 0.01), and the phenotype of the complemented strain *ΔsrtA/srtA* was partially restored to the wild type level (Figure 2A). Furthermore, the wild type strain displayed a significant increase in autoaggregation ability at pH 4.2 compared to that at pH 7.4 (*p* < 0.01), while the *srtA* mutant displayed only a slight increase (Figure 2A). These results suggest that the autoaggregation of *L. gasseri* Kx110A1 is sortase A dependent and is facilitated by acidic conditions.

Next, we investigated the hydrophobicity and the surface charge using a microbial adhesion to solvents (MATS) assay (Figure 2B). The high level of adhesion to the acidic solvent chloroform (approximately 95%) and the low level of adhesion to the basic solvent ethyl acetate (approximately 25%) observed with the wild type strain, suggested a nonacidic charge and poor electron acceptor property of *L. gasseri* Kx110A1. The wild type strain was highly hydrophobic (62% adhesion to xylene), while the adhesion of the *srtA* mutant to xylene was significantly reduced to 17% (*p* < 0.0001). The affinity of the complemented *ΔsrtA/srtA* strain for chloroform and xylene was partially restored that of the wild type strain. There were no significant differences in the affinity for ethyl acetate among strains. These data suggest that the surface hydrophobicity and the positive charge of *L. gasseri* Kx110A1 are influenced by SDPs anchored by sortase A-dependent mechanisms.

### Sortase A is Involved in the Adhesion of *L. gasseri* Kx110A1 to Gastric Epithelial Cells

**Figure 3:**
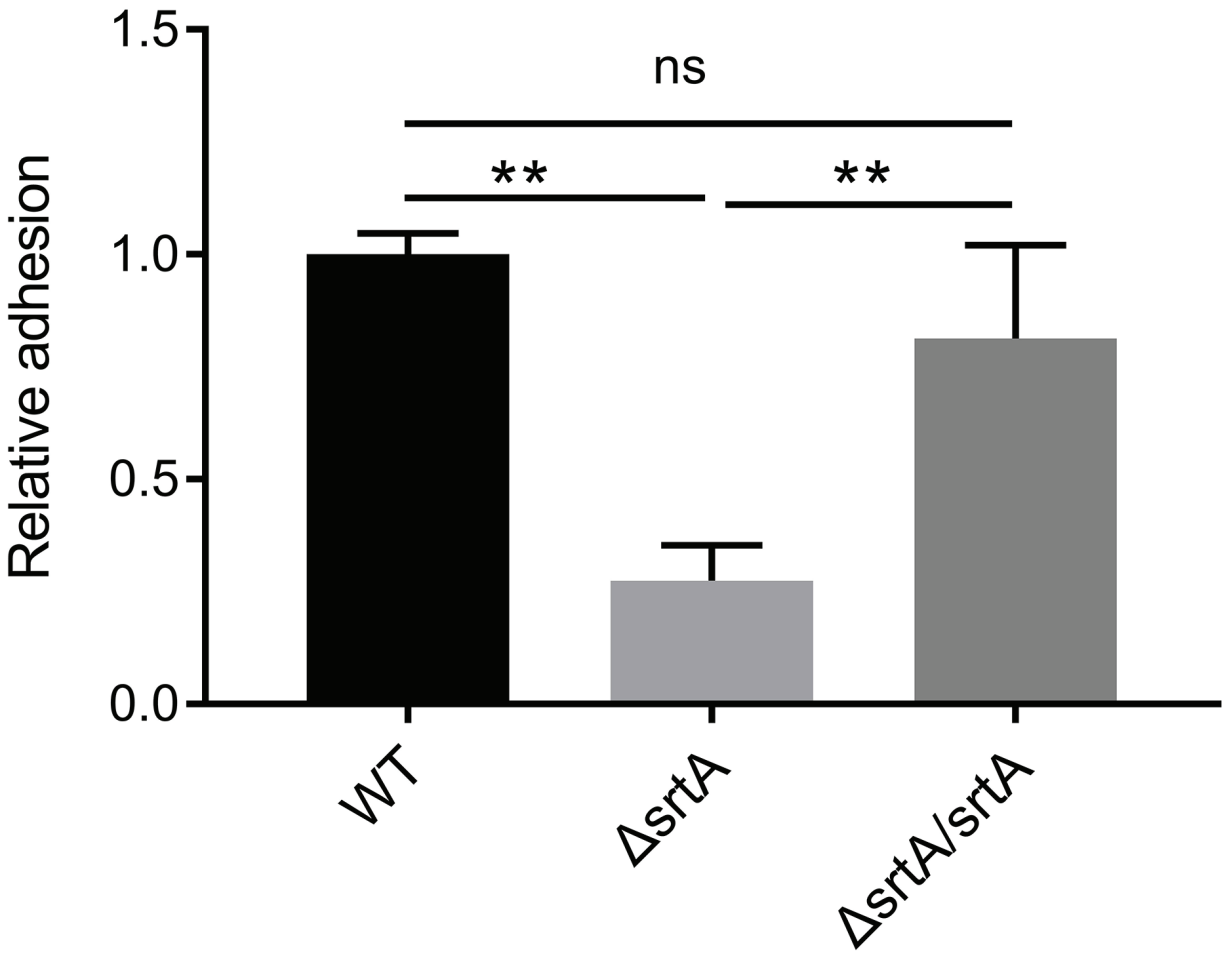
SrtA contributes to the adhesion of *L. gasseri* Kx110A1 to human gastric epithelial AGS cells. Adherence of *L. gasseri* Kx110A1 and its derivatives to AGS cells. Data represent the means ± SD of three independent experiments, **p < 0.01, ns, no significance.

The adhesion of wild type *L. gasseri* Kx110A1 and its derivatives to the gastric epithelial cell line AGS was measured. A significant 70% decrease in adhesion to the AGS cell line was observed in the *srtA* mutant compared to that of the wild type strain (*p* < 0.01) (Figure 3). The complemented strain has a similar adhesion ability as the wild type strain. This finding suggests that SDPs anchored by sortase A contribute to *L. gasseri* Kx110A1 adhesion to gastric epithelial cells.

### Sortase A Contributes to the Coaggregation of *L. gasseri* Kx110A1 With *H. pylori*

**Figure 4:**
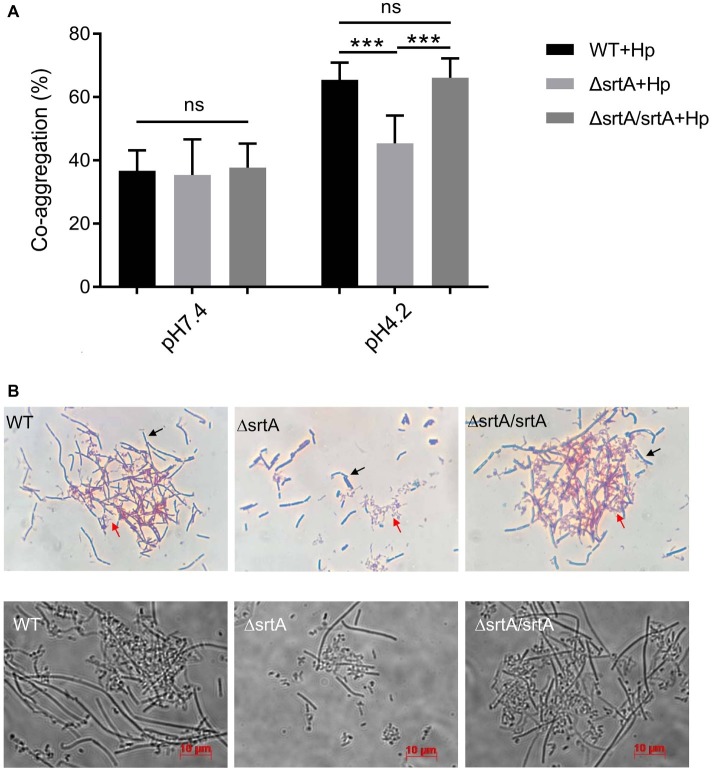
SrtA contributes to the coaggregation of *L. gasseri* Kx110A1 with *H. pylori* 67:21. **(A)**. Coaggregation of *L. gasseri* Kx110A1 with *H. pylori* 67:21 at pH 7.4 and pH 4.2. **(B)** The coaggregates were Gram-stained and subsequently observed under a light microscope at 100× (upper panel) or directly observed under a live-cell microscope (lower panel). Representative images are shown. The black arrow indicates lactobacilli, and the red arrow indicates *H. pylori*. Data represent the means ± SD of three independent experiments, ^***^p < 0.001, ns, no significance.

We further investigated the coaggregation ability of *L. gasseri* Kx110A1 with *H. pylori* 67:21 by spectrophotometry and microscopy. As expected, there were no significant differences among strains regarding the coaggregation with *H. pylori* at pH 7.4 (Figure 4A, left part). At pH 4.2; however, the *srtA* mutant displayed a significantly (*p* < 0.001) reduced ability to coaggregate with *H. pylori* 67:21 compared to that of wild type and the complemented strains (Figure 4A, right part). To determine whether the observed difference was a result of the autoaggregation of only lactobacilli instead of the coaggregation of lactobacilli and *H. pylori*, we checked the autoaggregation ability of *H. pylori* 67:21 at pH 4.2 (Figure 2A). The result showed that *H. pylori* 67:21 does not autoaggregate at pH 4.2, suggesting that *L. gasseri* Kx110A1 interacts with *H. pylori* and that sortase A-anchored SDPs contribute to this interaction.

We next investigated the direct interaction between *L. gasseri* Kx110A1 and *H. pylori* 67:21 by Gram staining and microscopy. The *L. gasseri* Kx110A1 wild type and complemented strain, but not the *srtA* mutant, formed large coaggregates with *H. pylori* 67:21 (Figure 4B). It seemed that *H. pylori* 67:21 bound to *L. gasseri* Kx110A1 and was trapped in the aggregates. These results suggest that SDPs contribute to the coaggregation of *L. gasseri* Kx110A1 with *H. pylori* 67:21.

### Sortase A Mediates the Reduction in *H. pylori* Adhesion to Gastric Epithelial Cells by *L. gasseri* Kx110A1

**Figure 5:**
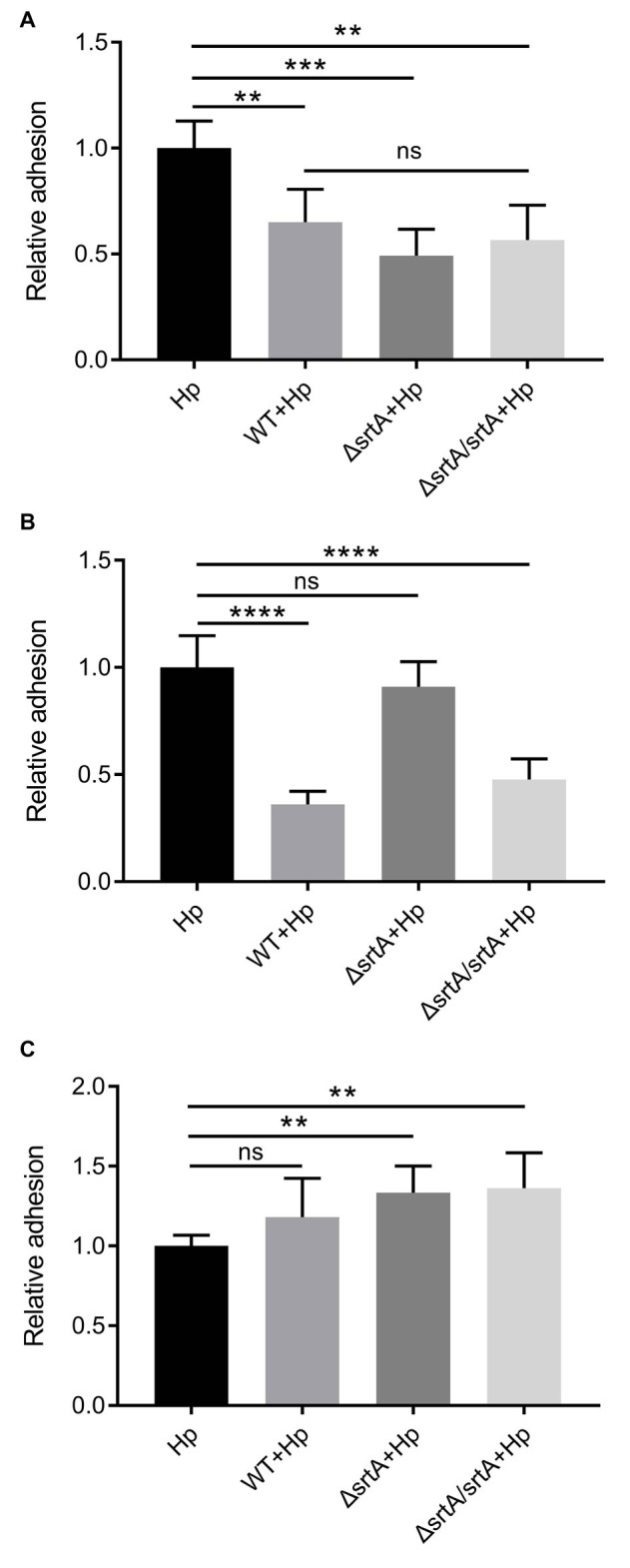
Inhibition of *H. pylori* adhesion to human gastric epithelial cells by *L. gasseri* Kx110A1. Adherence of *H. pylori* 67:21 to AGS cells in **(A)** a competition assay, both strains added simultaneously; **(B)** exclusion assay, lactobacilli pre-adhered for 2 h, then *H. pylori* added; **(C)** replacement assay, *H. pylori* pre-adhered for 2 h, then lactobacilli added. Data represent the means ± SD of three independent experiments, ^**^p < 0.01, ^***^p < 0.001, ^****^p < 0.0001, ns, no significance.

Competition, exclusion, and displacement assays were performed to further investigate the ability of *L. gasseri* Kx110A1 and its derivatives to reduce the adhesion of *H. pylori* 67:21 to AGS cells. The wild type, *srtA* mutant and complemented strains significantly reduced the adherence of *H. pylori* 67:21 by approximately 50% in a competition assay when both strains were added simultaneously (Figure 5A). Notably, wild type *L. gasseri* Kx110A1 significantly inhibited *H. pylori* 67:21 adherence by approximately 75% when preincubated with the host cells for 2 h before the addition of *H. pylori* in an exclusion assay (Figure 5B). However, the inhibition ability was completely lost in the *srtA* mutant, whereas the phenotype was restored to the wild type level in the complemented strain. In contrast, neither wild type *L. gasseri* Kx110A1 nor its derivatives were able to displace the pre-adhered *H. pylori* 67:21 in the displacement assay (Figure 5C). These data suggest that pre-colonization of *L. gasseri* Kx110A1 can prevent the initial adherence of *H. pylori* to gastric epithelial cells, which partially depends on the SDPs anchored by sortase A.

## Discussion

Adherence is a critical first step in the process of establishing mucosal infection for many pathogens, including *H. pylori*. Therefore, the inhibition of *H. pylori* adherence to gastric epithelial cells is important for preventing infection by this bacterium. Lactobacilli are among the most common bacteria in the stomach and display antagonistic activities against *H. pylori*, such as through competition for adhesion or coaggregation with the pathogen. For instance, the gastric isolate *Lactobacillus fermentum* UCO-979C forms a biofilm and thereby prevents the adhesion of *H. pylori* to gastric epithelial cells (Salas-Jara et al., 2016). *Lactobacillus reuteri* DSM17648 selectively coaggregates with *H. pylori* strains but not other commensal oral and intestinal bacteria (Holz et al., 2015). However, the inhibitory mechanisms and the effector molecules from lactobacilli that confer such a protective role are poorly understood (Bergonzelli et al., 2006).

Our previous study showed that the human stomach *L. gasseri* isolate Kx110A1 can colonize the mouse stomach and reduce the initial colonization of *H. pylori* (de Klerk et al., 2016). In this study, we aimed to investigate the role of SDPs, an important group of cell surface proteins in gram-positive bacteria, in *L. gasseri* Kx110A1 adhesion to gastric epithelial cells and competitive exclusion of *H. pylori*. To achieve this aim, an *srtA* knockout mutant of *L. gasseri* Kx110A1 was constructed, and the comparative functions were analyzed. Previous studies suggested that inactivation of sortase does not cause significant alterations in growth in lactobacilli (Malik et al., 2013; Call et al., 2015). However, the deletion of *srtA* leads to a slight growth defect in *L. gasseri* Kx110A1, suggesting that sortase A might couple certain SDPs to the cell wall, which is important for nutrient acquisition from the medium. Such a role for sortase A has been demonstrated in several *L. lactis* strains, in which proteinase P (PrtP) is a substrate of sortase A responsible for nutrient acquisition in a dairy environment (Vos et al., 1989; Dieye et al., 2010).

We further showed that the autoaggregation capacity of *L. gasseri* Kx110A1 is determined by sortase A. However, this capacity differs between *Lactobacillus* strains of other origins, such as *L. plantarum* CMPG5300 isolated from the human vaginal tract (Malik et al., 2013) and *Lactobacillus acidophilus* NCFM isolated from the intestinal tract (Goh and Klaenhammer, 2010), which show autoaggregation abilities in a neutral environment. The autoaggregation of *L. gasseri* Kx110A1 was observed under low pH conditions, suggesting that *L. gasseri* Kx110A1 is well adapted to the acidic stomach niche. In addition, the SDPs anchored by sortase A induced a nonacidic and hydrophobic cell surface of *L. gasseri* Kx110A1, as deletion of *srtA* dramatically reduced the hydrophobicity and affinity for chloroform. In contrast, SDPs might not contribute to the nonacidic nature of *Lactobacillus casei* BL23, as deletion of four different sortase-encoded genes in this strain did not alter its affinity for chloroform (Munoz-Provencio et al., 2012).

The cell wall-associated characteristics of lactobacilli, such as cell surface hydrophobicity and autoaggregation, are closely related to their adhesion properties (Kos et al., 2003). We further investigated the adhesion of *L. gasseri* Kx110A1 and its derivatives to AGS gastric epithelial cells. The sortase A mutant showed a significant reduction in adhesion to AGS cells. To our knowledge, this is the first data showing an important role of sortase and SDPs in the adhesion of lactobacilli to gastric epithelial cells. Several mutagenesis studies have demonstrated the role sortase and SDPs play in the adhesion of lactobacilli, such as *L. plantarum* (Pretzer et al., 2005; Malik et al., 2013), *L. casei* (Munoz-Provencio et al., 2012), *L. acidophilus* (Call et al., 2015), *Lactobacillus gasseri* (Call et al., 2015; Arai et al., 2016), *Lactobacillus salivarius* (van Pijkeren et al., 2006), *L. reuteri* (Jensen et al., 2014), and *Lactobacillus johnsonii* (Denou et al., 2008), to intestinal or vaginal epithelial cell lines. Our results further provide evidence that sortase and SDPs are involved in shared probiotic mechanisms among different *Lactobacillus* species (Call and Klaenhammer, 2013; Sanders et al., 2018).

We investigated the role of sortase A and SDPs in the ability of *L. gasseri* Kx110A1 to antagonize *H. pylori*. Coaggregation has been described to be a potential mechanism for preventing pathogens from binding or invading host cells (Reid et al., 1990; Younes et al., 2012; García-Cayuela et al., 2014). We found that the ability of *L. gasseri* Kx110A1 to coaggregate with *H. pylori* is highly related to its autoaggregation, as it also showed pH dependence and was dependent on sortase A. *H. pylori* has been shown to bind to cell surface molecules of lactobacilli, such as the chaperone GroEL (Bergonzelli et al., 2006) or other unknown surface molecules (Holz et al., 2015). It is difficult to determine whether *H. pylori* directly binds to any SDPs anchored by sortase A at the cell surface of *L. gasseri* Kx110A1 since binding was also detected in the mutant. However, the pathogen was frequently trapped inside aggregates, suggesting that the coaggregation observed in this study might be the result of the autoaggregation ability of *L. gasseri* Kx110A1. *Lactobacillus* coaggregation with *H. pylori* might also allow the pathogen to be removed by the host body fluids (Spurbeck and Arvidson, 2010). In addition, pre-colonization of *L. gasseri* Kx110A1 wild type and the complemented strain, but not the sortase A mutant, inhibited the adherence of *H. pylori* to host epithelial cells. The inability of the mutant to prevent attachment of *H. pylori* is most likely due to the reduced adherence of the mutant strain compared to that of the wild type strain, thus leaving space for *H. pylori* to adhere. Adherent lactobacilli have been shown to competitively exclude pathogens by successfully preventing or limiting the adhesion of the pathogen to host epithelial cells and mucus (Bermudez-Brito et al., 2012). Our results suggest that sortase A-anchored SDPs in lactobacilli competitively exclude *H. pylori* by means of steric hindrance rather than by specific blockage of receptor sites, which has been demonstrated in many studies (Chan et al., 1985; Coconnier et al., 1993; Zarate and Nader-Macias, 2006). Steric hindrance appears to be the predominant mechanism by which lactobacilli antagonize pathogens *in vivo* (Charteris et al., 1998). Interestingly, wild type *L. gasseri* Kx110A1 and its derivatives showed a similar level of adherence inhibition when lactobacilli were added to the gastric epithelial cells in combination with *H. pylori*. We speculate that another mechanism occurred in this model of treatment, i.e., an effector molecule secreted by *L. gasseri* Kx110A1 may reduce *H. pylori* adherence by inhibiting the expression of the adhesion gene *sabA* (de Klerk et al., 2016), while sortase A does not contribute to this mechanism (unpublished data). However, the role of sortase A cannot be completely ruled out since *H. pylori* trapped in the *L. gasseri*-*H. pylori* coaggregates were also enumerated in the adhesion assays.

To further determine which SDPs may be involved in the probiotic features of *L. gasseri* Kx110A1, 13 putative SDPs were identified. However, the expression of SDP-encoding genes remained unaffected in the sortase A mutant, which is consistent with a previous study in *L. plantarum* (Remus et al., 2013). This finding could be explained by the fact that SDPs may not be coupled covalently to the peptidoglycan in the absence of sortase activity, while they are still produced and remain loosely associated with the cell surface (Nobbs et al., 2007; Remus et al., 2013). The exact SDPs involved in the autoaggregation and adhesion of *L. gasseri* Kx110A1 remain to be investigated in future studies.

Taken together, our results show that sortase A is a key determinant of the surface properties of *L. gasseri* Kx110A1 and contributes to the *Lactobacillus*-mediated inhibition of *H. pylori* attachment to host cells. To the best of our knowledge, this is the first report that demonstrates such a role of sortase A in a *L. gasseri* strain of human stomach origin. A better understanding of the molecular mechanisms underlying the antagonistic effect of lactobacilli will contribute to the practical applications for the prevention and treatment of *H. pylori* infection.

## Data Availability Statement

All datasets generated for this study are included in the article/Supplementary Material.

## Author Contributions

FZ and A-BJ conceived the study. FZ designed the experiments. FZ, AA, and HG performed all the experiments. FZ and A-BJ wrote the manuscript. All of the authors read and approved the final manuscript.

### Conflict of Interest

The authors declare that the research was conducted in the absence of any commercial or financial relationships that could be construed as a potential conflict of interest.
