# Supplementary material for: Role of Sortase A in Lactobacillus gasseri Kx110A1 Adhesion to Gastric Epithelial Cells and Competitive Exclusion of Helicobacter pylori
Source: Front Microbiol. 2019 Dec 3;10:2770. doi: 10.3389/fmicb.2019.02770 (PMC6902081; doi:10.3389/fmicb.2019.02770)

Supplementary material

Table S1 Primers used in this study

Primers	Sequence (5'-3') [a]	Restriction site	Purpose
srtA-F	AAAAATGAATGATGACGAT		Sequencing
srtA-R	CATTTATAACATCTAAGTTTCTAT		Sequencing
srtA-up-F	ACTTCGAGCTCATTAGGTTTTGGCTTCCGTT	*Sac*I	Cloning
srtA-up-R	TTTTTGGATCCATTGTCTTTGTCCTCAATTTTATT	*BamH*I	Cloning
srtA-down-F	AACTTGGATCCAGAGGATTTGCTTTTGTTGAAG	*BamH*I	Cloning
srtA-down-R	CATCAGCATGCAAAAGTAGTTCAACCGCTAAACT	*Sph*I	Cloning
pIrecSC-F	GGAATTGTCAGATAGGCCTAATGACT		Cloning
pIrecSC-R	AAAGTTTTCGGGCTACTCTCTCCT		Cloning
srtAseq-F	AGTTTGAACCTGAAACTTCTCAAGC		Cloning
srtAseq-R	CTTCAGCCTGTTCTTCATCATCACT		Cloning
comSrtA-F	ATGGCAGATCTAAAAATGAATGATGACGAT	*Bgl*II	Cloning
comSrtA-R	TTCACCTCGAGCATTTATAACATCTAAGTTTCTAT	*Xho*I	Cloning
srtArt-F	ATCGGTTTAGGGATGATTTTTA		qPCR
srtArt-R	CATTCCTTGTTTTGCTTGATT		qPCR
lepArt-F	TCGTGCGACAATTAAACCATAT		qPCR
lepArt-R	CTTCTACCTTTCCGACTGCCT		qPCR
recJrt-F	TGGAAAAGTCTATTAACCGCAT		qPCR
recJrt-R	AAAAGTGAACATCGGCACCT		qPCR
gyrB-F	TTTGCTAACAACATTCACACCT		qPCR
gyrB-R	ACGAATATCCTCACCAGATAAGT		qPCR
LGAS_1725-F	AGCAGATAAAGTAGAGCAGAATG		PCR/qPCR
LGAS_1725-R	GTACCAAGTTGTTTTTGAGATGT		PCR/qPCR
LGAS_1671-F	AAGGTGAAAATGTAGAAGCTGAT		PCR/qPCR
LGAS_1671-R	TGGTATTTGGTGTGTTAGCATT		PCR/qPCR
LGAS_0942-F	AATAAAGAAGGGACAAGCTACAT		PCR/qPCR
LGAS_0942-R	GCTGGTCTACCTAACAATGGAT		PCR/qPCR
LGAS_0943-F	AGCAGAATCAACTGAACAACAA		PCR/qPCR
LGAS_0943-R	TAAACCAAGTAAACCACCAACA		PCR/qPCR
LGAS_0143-F	CCATCTGAATCTGGCGTTTAT		PCR/qPCR
LGAS_0143-R	ATTGTCTTACCGACTTGCTTATT		PCR/qPCR
LGAS_0146-F	TGGTGGTCAAGCAACTACTAAAC		PCR/qPCR
LGAS_0146-R	AGCAATACCAACTGAAGCAACT		PCR/qPCR
LGAS_0410-F	AAGAAGCTGAGGAGAGTCCACT		PCR/qPCR
LGAS_0410-R	AATCCACCTAACTTTTGCGTAAT		PCR/qPCR
LGAS_1067-F	TTACAAGCAACTATTTAGTGGAT		PCR/qPCR
LGAS_1067-R	AACTTACGATTTAATTCAGGAG		PCR/qPCR

LGMS_0331-F	ACTTCATCGCTCACTTTACTCAT	PCR/qPCR
LGMS_0331-R	TTGACCTTTCTTAGGACCATTT	PCR/qPCR
LGMS_0851-F	CATACCTGATTTTGTCAACCATT	PCR/qPCR
LGMS_0851-R	TAATTTGCCTTTTCATATATACACT	PCR/qPCR
LGMS_0790-F	TAGTAGAACGACATAATGAGCAAT	PCR/qPCR
LGMS_0085-F	TTCAATAGCTGTTGGGTGTTT	PCR/qPCR
LGMS_0085-R	TTTAACTTGGTTTGGATCTTGT	PCR/qPCR
LGAS_0045-F	AAAATGATGAACAAAATAAGCAA	PCR/qPCR
LGAS_0045-R	TTTAGACGCACTAAAAGAAGCTA	PCR/qPCR
LGAS_0383-F	CTAAGAAAGAAGCAGTACGTGTT	PCR/qPCR
LGAS_0383-R	TTTGAAGCAATTCTGTTAGTAAGT	PCR/qPCR
LGAS_0866-F	TGAAAAGAATGTCAGCGTAGTA	PCR/qPCR
LGAS_0866-R	AACTGCACTAATTGCTGCTACT	PCR/qPCR
LGAS_1663-F	CTGATGAAAAGTCAGGCTATACA	PCR/qPCR
LGAS_1663-R	CTAATCGAAGACAGATGATGGAT	PCR/qPCR

[a] The restriction site is underlined in primer sequence.

Table S2 Putative SDPs identified in the genome of *L. gasseri* Kx110A1.

Locus tag	Predicted function	Signal peptide (+/-)	LPXTG motif	Presence in *L. gasseri* Kx110A1 (+/-) [a]
LGAS_0942	Adhesion exoprotein	+	LPQTG	+
LGAS_0943	Adhesion exoprotein	-	LPQTG	+
LGAS_0143	Adhesion exoprotein	-	LPQTG	+
LGAS_0410	Adhesion exoprotein	-	LPQTG	+
LGAS_0045	Adhesion exoprotein	-	LPQTG	+
LGMS_0851	Cell division membrane protein	-	LPITG	+
LGMS_0790	Cell division membrane protein	-	LPLTG	+
LGAS_0146	DNA polymerase elongation subunit	+	LPQTG	+
LGMS_0331	Conserved hypothetical protein	+	LPQTG	+
LGAS_0383	hypothetical protein	-	LPQTG	-
LGAS_0866	hypothetical protein	+	LPETE	+
LGAS_1663	hypothetical protein	+	LPQTG	-
LGAS_1067	5'-nucleotidase/2',3'-cyclic phosphodiesterase related esterase	+	LPKAG	+
LGMS_0085	Beta-N-acetylhexosaminidase	+	LPKTG	-

[a] Determination of the presence of the possible SDPs in *L. gasseri* Kx110A1 based on PCR amplification of indicated genes by specific primers that designed according to SDPs indentified in *L. gasseri* strains ATCC33323 and SBT2055.

Fig. S1. Transcripts analysis of the two flanking genes of *srtA* in the *L. gasseri* Kx110A1 wild type, *srtA* mutant, and complemented strains by qPCR.

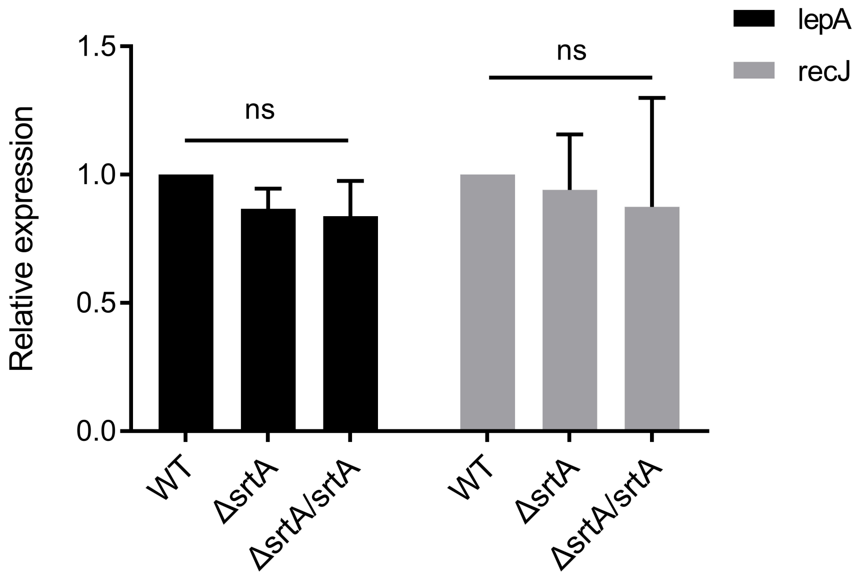

Fig. S2. Transcripts analysis of the genes encoding putative SDPs in *L. gasseri* Kx110A1 wild type, *srtA* mutant, and complemented strains by qPCR.

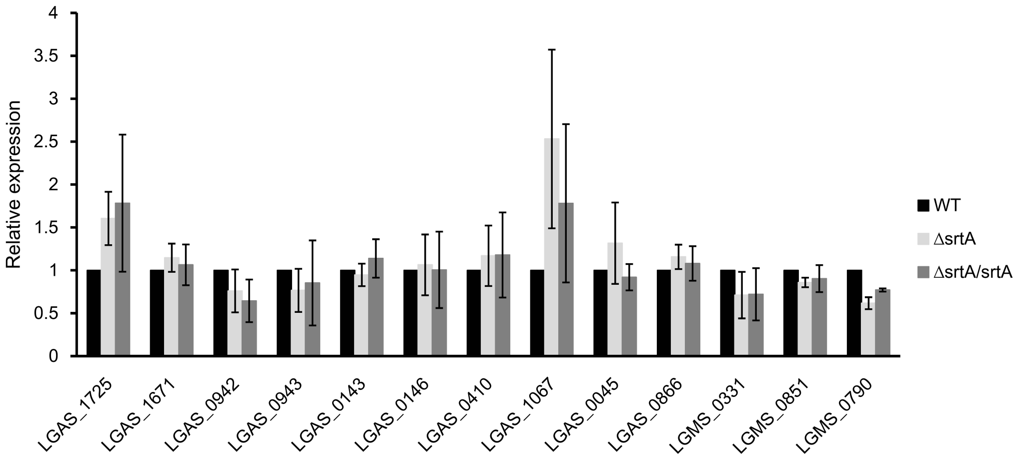

Fig. S3. Phenotypes of *L. gasseri* Kx110A1 wild type, sortase A mutant, and complemented strains. (A) Growth curve of strains cultivated in MRS medium. (B) Overnight cultures of the wild-type, *srtA* mutant, and complemented strains in MRS medium. (C) Single colonies on Rogosa agar plates.

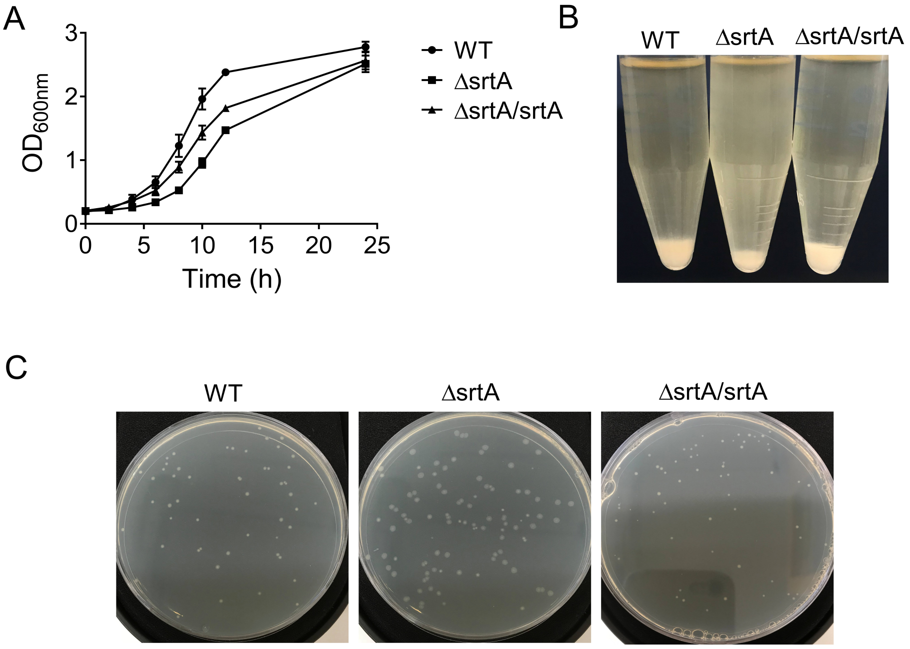

Supplement: Supplementary file 1 [file Data_Sheet_1.pdf]
